# Research-based education in undergraduate occupational therapy and physiotherapy education programmes: a scoping review

**DOI:** 10.1186/s12909-022-03354-2

**Published:** 2022-05-11

**Authors:** Kjersti Velde Helgøy, Tore Bonsaksen, Kari Røykenes

**Affiliations:** 1 Center of Diakonia and Professional Practice, VID Specialized University, Oslo, Norway; 2 Department of Health and Nursing Science, Faculty of Social and Health Sciences, Inland Norway University of Applied Science, Elverum, Norway; 3 Department of Health, Faculty of Health Studies, VID Specialized University, Sandnes, Norway; 4 Faculty of Health Studies, VID Specialized University, Bergen, Norway

**Keywords:** Research-based education, Evidence-based practice, Occupational therapy education, Physiotherapy education, Undergraduate education

## Abstract

**Background:**

In a research-based learning environment, students learn how to become critical thinkers and lifelong learners, and to generate discipline-enriching knowledge. Research training is important for all healthcare professionals and the integration of research in education can be one approach to improve evidence-based practice among future professionals. The purpose of this scoping review was to identify studies reporting on research-based education in undergraduate occupational therapy and physiotherapy curricula to document the current state of knowledge and to map factors that reflect and support the implementation of research-based education in undergraduate occupational therapy and physiotherapy programmes.

**Methods:**

A scoping review was used to systematically select and summarize existing literature. The search was conducted using a combination of keywords and MeSH terms in the following databases: EBSCO (Academic Search Elite, CINAHL, ERIC), MEDLINE, Embase, Education Source and grey literature. A thematic analysis identified strategies used to implement and promote research-based education in occupational therapy and physiotherapy bachelor programmes.

**Results:**

The database search identified 3068 records. 75 were eligible for full-text assessment and 27 studies were included. The studies were published between 1999 and 2021 and were conducted in Norway, Canada, South Africa, Australia, the United Kingdom, the United States, New Zealand, Ireland, Taiwan, Italy, and Iran. The study designs were mainly quantitative and qualitative, and studies included primarily occupational therapy and physiotherapy students. We identified the following four strategies: ‘student engagement in research’, ‘curriculum improvement regarding EBP’, ‘EBP teaching’ and ‘journal club’, where ‘EBP teaching’ was most frequently identified.

**Conclusions:**

Findings from this scoping review suggest that ensuring students’ competence in research methods is necessary for students to be able to read and understand research articles, which are important as foundational skills in undergraduate research training. Journal clubs can be a foundation for student engagement with research literature, and students’ basic research skills may be facilitated through their involvement in research projects and by conducting systematic reviews. Further, cooperation with clinical practice is important, and the awareness of research-based education should be increased among both faculty members and students.

**Supplementary Information:**

The online version contains supplementary material available at 10.1186/s12909-022-03354-2.

## Background

In a research-based learning environment, students learn about research processes by developing research skills, such as the ability to critically analyse and reflect [1]. Research skills are necessary for future professionals, as previous studies have indicated that implementing clinical guidelines and research evidence into routine daily practice is challenging [2, 3]. Based on the Sicily statement on evidence-based practice (EBP), all healthcare professionals need to adopt a critical stance towards their own practice and the underlying evidence [4]. Without such a critical stance, professionals will be unable to provide ‘best practice’. Professional education is believed to play an important role in the development of positive attitudes towards EBP skills [5, 6]. One approach to improving EBP uptake in clinical practice is through the integration of research in education [7, 8].

The link between research and teaching in higher education has been explored in several studies [1, 9–19]. One strategy for linking research and teaching is to bring research into the classroom, e.g., through academics presenting their research relevant to the subject and discussing research outcomes and methods with students [1]. Different models of research-based education currently exist, and in this study we have used Huet’s model [1]. We included this model to guide our study since it is more recent and represents a further development of previous models within research-based education. This model uses the terms research-based teaching and research-led teaching. Research-based teaching occurs when students are engaged in research or research-related tasks, while research-led teaching occurs when teachers use their competence as researchers, or use research conducted by others, to inform teaching. In this model these terms are connected, and research and teaching are described as two integrated parts.

Previous studies have described strategies to support faculty in integrating research and inquiry in the curriculum [20, 21]. Engaging students in research and inquiry from the beginning of their first study year has been recommended [20]; moreover ensuring coherence in curriculum planning as well as systematic thinking regarding how students can obtain skills to engage in research — whether that is reading research articles, taking part in research methods courses or participate in research projects — seems important [21]. Huet [1] has suggested four actions regarding the implementation of research-based education in the curriculum. First, clear guidelines for embracing a research-based education model are needed. Second, the time needed to conceptualize, plan and deliver research-based and research-led teaching approaches must be considered. Third, research and teaching should be considered as two integrated activities. Fourth, pedagogic support needs to be provided to develop learning and teaching strategies that are sustained in research-led or research-based approaches in the early stages of the programme. Based on these recommendations, it seems beneficial to embrace a research-based education model in the education programme, and to integrate this way of linking research and teaching in the organization of that programme [1].

Research-based undergraduate education has received increased attention in countries such as the United States, the United Kingdom, Australia and Sweden [22]. In Norway, it is required that higher education is research-based [21]. According to the relevant legislation, education must be on the cutting edge in terms of research and development work [21]. Accreditation of health profession education programmes also has relevance for discussions regarding research-based education, as it is concerned with quality within teaching and research [23]. It has been argued that accreditation is essential to a profession as it ensures that students who have graduated are competent to practice safely and effectively [23]. To ensure research-based education, educators research competence is significant. Hence, in some countries, it is not uncommon to expect that approximately 25% of faculty members hold a PhD. In Norway, 20% of the faculty members teaching in bachelor programmes must hold a PhD or equivalent [24]. Research-based education has also been emphasized in a recent White Paper on quality in higher education, where research-based education is defined as education that is linked to a research environment; is conducted by staff who also carry out research; builds on existing research in a particular field; provides knowledge about the philosophy of science and research methods; and provides opportunities for students to learn how research is conducted from staff or students themselves conducting research as part of their studies [25]. This definition has some similarities with Huet’s [1] definition of research-based education, such as describing elements of both research-based and research-led education; however, the White Paper provides additional examples.

As such, research-based education and EBP are different concepts. Research-based education consists of an educational environment where academics and students teach and learn through inquiry and research [1], while EBP is concerned with using the best evidence from high-quality studies with a clinician’s expertise and patient’s preferences and values in the provision of healthcare [26]. Research-based education can take various forms, such as developing research skills through learning about research and research methods and taking part in research projects [21]. Further, it may vary according to the discipline [21]. In occupational therapy education, for example, learning outcomes have been described as ‘the candidate should have knowledge about EBP and knowledge about philosophy of science, research ethics and research methods, and how this has significance for knowledge development in occupational therapy’ [27]. Teaching methods related to EBP have relevance for research-based education, as EBP emphasizes that research should underpin clinical decisions. For professionals in occupational therapy and physiotherapy, EBP skills are important. Health-care graduates should be able to gain, assess, apply and integrate new knowledge, and one approach to achieve this is to train students in developing these skills through the five-step model of EBP [4]. Previous reviews have investigated teaching strategies focusing on EBP among various health educations [28–33]. Results indicate that EBP teaching in nursing should include knowledge transfer to clinical situations [29, 31]; moreover, information literacy is considered among the most important competences for EBP [29], and a combination of lectures, seminars, discussion, exams, assignments, small-group work, team-based learning, case-study analysis and individual learning are recommended [30]. In previous reviews, multifaceted approaches—including lectures, tutorials, workshops and journal clubs—have also been found to be best-suited for teaching EBP to health students from programmes including medicine, physiotherapy and occupational therapy [32, 33]. However, the majority of the included studies were not conducted among occupational therapy and physiotherapy students. Further, research courses, workshops and collaboration with clinical practice are recommended methods for teaching EBP to undergraduate healthcare students [28].

Compared to studies focusing on teaching strategies in EBP, fewer studies have investigated strategies to implement and support research-based education across healthcare programmes. However, one scoping review investigated research competencies among rehabilitation students, including physiotherapy and occupational therapy students [34]. Several core research competencies were identified in this study [34], and most fell within two research domains: inquiry/literature review and methodology/processes. Research competencies within the inquiry/literature review included recognizing gaps in the literature, searching for and locating relevant literature and critical evidence-appraisal skills. Within methodology/processes, research competencies such as knowledge of research design, data collection skills and data analysis skills were described. As the uptake and integration of research in education programmes is believed not only to improve students’ research skills but also to support their future use of research in practice, it is important to gain more knowledge about the ways in which research-based education is reflected and supported. Previous studies investigating research-based education are limited, especially in bachelor programmes in healthcare [22]. Studies concerned with occupational therapy and physiotherapy bachelor programmes seem particularly scarce compared to other programmes, and it is therefore important to gain more knowledge regarding research-based education in these education programmes. In this scoping review, the purpose was to identify studies reporting on research-based education in undergraduate occupational therapy and physiotherapy curricula to document the current state of knowledge. A further objective was to map factors that reflect and support the implementation of research-based education in undergraduate occupational therapy and physiotherapy programmes.

## Methods

A scoping review was conducted to identify studies reporting on research-based education in occupational therapy and physiotherapy curricula, and to map factors that reflect and support the implementation of research-based education in these programmes. We followed the framework for scoping reviews developed by Arksey and O’Malley [35] and further developed by Levac et al. [36] and Khalil et al. [37]. We used a protocol based on Arksey and O’Malley’s framework [35] to address each of the five steps for undertaking a scoping review: 1) identifying the research question; 2) identifying relevant studies; 3) selecting studies; 4) charting the data; and 5) summarizing and reporting the results. The Joanna Briggs Institute Manual for Evidence Synthesis (hereafter, the JBI manual) was also used throughout the scoping review process to ensure a systematic methodology [38].

### Step 1: identifying the research question

The objective of the current study was:

1. To explore the extent and range of research on research-based education conducted in occupational therapy and physiotherapy education.
2. To explore research methods and design used in research on research-based education in occupational therapy end physiotherapy education.
3. To explore strategies used to implement and promote research-based education in occupational therapy and physiotherapy education.

### Step 2: identifying relevant studies

As recommended in the JBI manual [38], the search strategy was developed in consultation with a research librarian. The search strategy was reviewed by another research librarian before the search was conducted. This was an iterative process. Publications in English, Norwegian, Swedish and Danish published between 1 January 1990 to 15 June 2021 were included in this review. The searches were conducted in the following databases: EBSCO (Academic Search Elite, CINAHL, ERIC), MEDLINE, Embase and Education Source, using a combination of keywords and MeSH terms. These databases cover both health and education. Grey literature was searched for in Nora, Bibliotek DK, Libris, Norart, SveMed+, Bielefeld Academy Search Engine, CADTH, Current Awareness Service for Health, Copac, CORE, EThOS, King’s Fund, MedNar, NDLTD, OAIster, Open Access Theses and Dissertations, National Institute for Clinical Excellence and OpenGrey. Table 1 provides a list of specific search queries used in all databases.

**Table 1:** Specific search queries used in all databases

Database	Search queries
ACADEMIC SEARCH ELITE	(Research skills OR inquiry-based learning OR research-based education OR research-based OR research-led OR research-oriented OR research evidence OR research skills OR evidence-based practice OR EBP OR research utilization OR inquiry-based learning) AND (occupational therapy education OR physical therapy education OR curriculum planning OR physiotherapy education OR curriculum development)
EDUCATION SOURCE	(Research skills OR inquiry-based learning OR research-based education OR research-based OR research-led OR research-oriented OR research evidence OR evidence-based practice OR EBP OR research utilization) AND (occupational therapy education OR physical therapy education OR curriculum planning OR physiotherapy education OR curriculum development)
CINAHL® with full text	(Professional practice, evidence-based OR learning methods OR research-based education OR research-based OR research-led OR research-oriented OR research evidence OR research skills OR evidence-based practice OR EBP OR research utilization OR inquiry-based learning) AND (education, occupational therapy OR education, physical therapy OR curriculum development OR occupational therapy education OR physical therapy education OR physiotherapy education OR curriculum development)
ERIC	(Research skills OR evidence-based practice OR research utilization OR research-based education OR research-based OR research-led OR research-oriented OR research evidence OR research skills OR evidence-based practice OR EBP OR research utilization OR inquiry-based learning) AND (curriculum development OR occupational therapy education OR physical therapy education OR physiotherapy education)
AMED (Allied and Complementary Medicine	(Research-based education OR research-based OR research-led OR research-oriented OR research evidence OR research skills OR evidence-based practice OR EBP OR research utilization OR inquiry-based learning) AND (occupational therapy education OR physical therapy education OR physiotherapy education OR curriculum development)
EMBASE	(Evidence-based practice OR research-based education OR research-based OR research-led OR research-oriented OR research evidence OR research skills OR evidence-based practice OR EBP OR research utilization OR inquiry-based learning) AND (occupational therapy education OR physical therapy education OR curriculum development OR physiotherapy education OR curriculum development)
OVID MEDLINE	(Evidence-based practice OR research-based education OR research-based OR research-led OR research-oriented OR research evidence OR research skills OR evidence-based practice OR EBP OR research utilization OR inquiry-based learning) AND (occupational therapy education OR physical therapy education OR physiotherapy education OR curriculum development)

### Step 3: selecting studies

The research librarian exported all search results into Rayyan, a web-based app for systematic reviews [39]. After the duplicates were excluded, two reviewers independently screened the titles and abstracts of the remaining articles for relevance to the research question and the inclusion/exclusion criteria. As recommended in the JBI manual [38], a pilot test was conducted by a research team before the included articles were read. In this pilot test, three full-text articles were read and assessed by three researchers, and full agreement among the researchers were reached. All full-text articles were then read and assessed. They were further checked for relevance by two independent reviewers. If there was any disagreement, a third reviewer was included in the process.

### Inclusion criteria

#### Population

Studies reporting on students and/or faculty members in occupational therapy and physiotherapy bachelor programmes were included. Studies with allied health students or faculty members were included if they also included occupational therapy and physiotherapy bachelor programmes.

#### Concept

Studies with research-based education as the main concept were investigated in this review. That is, studies were included if they emphasized research in education, such as students’ use of research, students’ learning of research skills (including research methods and philosophy of science), and student involvement in research. Further, included studies focused on use of educational strategies to promote and implement research in curricula. Studies investigating faculty members’ use of research in teaching and involvement in research projects were also included. Finally, included studies focused on EBP and research utilization in occupational therapy and physiotherapy bachelor programmes.

#### Context

Included studies involved typical educational settings, e.g., classroom teaching, clinical placement or simulation training.

### Exclusion criteria

Studies conducted among bachelor students in other health disciplines—such as nursing and radiography—were excluded. Studies including students from master’s degree education programmes were excluded, as were studies focusing on occupational therapy and physiotherapy clinicians. We also excluded studies presented in languages other than English, Danish, Norwegian or Swedish.

### Step 4: charting the data

We used a pre-defined extraction form to analyse the results, as recommended in the JBI manual [38] (see Supplementary file Data Abstraction Table). The selected articles were reviewed by two independent reviewers. The data extraction tools formed the basis for the final presentation of the results in Tables 2 and 3. Study characteristics included first author, year of publication, title, country of origin, study design/purpose, study participants, context and main study findings. We used thematic analysis inspired by Aveyard [40] to identify strategies for implementing and promoting research-based education in occupational therapy and physiotherapy bachelor programmes. In accordance with Aveyard’s thematic analysis, we first identified themes from the results section of each included study. We used different colours to mark the parts that had relevance for our research questions. Further, we developed our themes by merging results with the same theme. Next, we named our themes after assessing the results from the included studies again, and then compared the themes to ensure that the names were suitable. The last step was to examine the themes, looking for similarities and differences in the material. Further, we analysed these to determine whether there were any recommendations regarding *when* to include the strategy in the curriculum, and *where*—i.e., in clinical placements, in the classroom or a combination of the two.

## Results

### Literature search

**Fig. 1:**
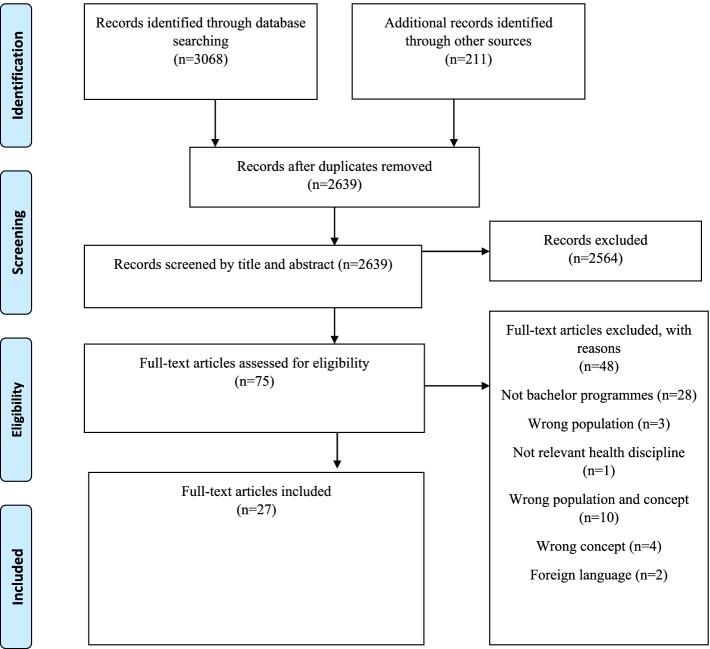
Modified PRISMA flow diagram illustrating the scoping review process

A total of 3068 records were identified in the following databases: ERIC (*n* = 595), Academic Search Elite (*n* = 315), AMED (*n* = 18), CINAHL with full text (*n* = 1311), Education Source (*n* = 388), Embase (*n* = 359) and MEDLINE (*n* = 82). The database search returned 2639 records after duplicates were removed. Further, grey literature was identified in 18 databases (*n* = 210). One article [34] was recommended by another researcher, and we included this article in the review after we checked it against the inclusion and exclusion criteria. The entire process of study identification and inclusion/exclusion is presented in Fig. 1.

#### Study characteristics

Study characteristics are presented in Table 2. Studies were spread across the years 1999–2021. The studies were conducted in Norway (*n* = 6), Canada (*n* = 5), South Africa (*n* = 5), Australia (*n* = 3), the United Kingdom (*n* = 2), the United States (*n* = 1), New Zealand (*n* = 1), Ireland (*n* = 1), Taiwan (*n* = 1), Italy (*n* = 1) and Iran (*n* = 1). The study designs were qualitative (*n* = 9), quantitative (*n* = 9), mixed methods (*n* = 2), review (*n* = 2), opinion piece (*n* = 1), model presentation (*n* = 1), curriculum design (*n* = 2) and designing and implementing teaching activities (*n* = 1). Study participants included physiotherapy students (*n* = 5), occupational therapy students (*n* = 5), allied health students (including occupational therapy and physiotherapy students; (*n* = 2), occupational therapy and physiotherapy students (*n* = 1), EBP teachers (*n* = 2), faculty members (*n* = 4), students and faculty members (*n* = 2), students, faculty members and clinical instructors (*n* = 2), occupational therapy students and clinicians (*n* = 1), occupational therapy education (*n* = 2) and physiotherapy education (*n* = 1).

**Table 2:** Study characteristics (*N* = 27)

First author and year of publication	Country	Title	Design/purpose	Participants	Context	Main study findings
Bannigang 2009 [41]	Canada	A Model of Professional Thinking: Integrating Reflective Practice and Evidence-Based Practice	A model presentation	Occupational therapy education	Classroom	Rather than teaching reflective practice and EBP as discrete subjects, ‘professional thinking’ should be taught as an overarching topic within curricula.
Boruff 2011 [42]	Canada	Integrating Evidence-Based Practice and Information Literacy Skills in Teaching Physical and Occupational Therapy Students	A librarian and a faculty member designed an instructional activity that included a lecture, workshop and assignment that integrated EBP skills in the first year of the programme	Occupational therapy and physical therapy students (*n* = 104)	Classroom	The teaching activities developed for the students in this course appear to have achieved the goal of teaching students the EBP research cycle so that they might begin to emulate it. The collaboration between the faculty member and the librarian was integral to the success of this endeavour.
Burger 2014 [43]	South Africa	Integrating Evidence-Based Principles Into the Undergraduate Physiotherapy Research Methodology Curriculum: Reflections on a New Approach	Qualitative	International experts	Classroom	Conducting a systematic review or meta-analysis to teach undergraduate students research methodology provides the opportunity for physiotherapy students to acquire basic research skills and become clinicians who provide EBP services.
Charumbira 2021 [34]	South Africa	Research Competencies for Undergraduate Rehabilitation Students: A Scoping Review	Scoping review	Physiotherapy, occupational therapy and speech and language therapy students	Classroom	A set of 45 research competencies were identified. It remains unclear which of these should be prioritized in the rehabilitation curriculum. This set may guide future consensus statements and allow educators to identify gaps in the curriculum.
Chia 2012 [44]	United Kingdom	What’s in a Journal Club?	Qualitative Grey literature	Occupational therapy students	Classroom	Participation in journal clubs at an early stage provides a foundation for engagement with professional literature and the recognition of the importance of EBP.
Da Roit 2016 [45]	Italy	Undergraduate Physiotherapy Students’ Competencies After a Reorganization of the Educational Pathways in EBP: Time and Type of Activity Are Relevant?	Quantitative comparative design (between first-, second- and third-year students) in physiotherapy Grey literature (paper presented at the 4th European Congress of the ER-WCPT/Physiotherapy)	Physiotherapy students (*n* = 101)	Classroom	A new multi-modal educational pathways was less effective in educating students in EBP; main reasons may be the reduction in amount of time dedicated to EBP, the lack of journal club activities and the lack of a formal assessment in the first year.
Du Toit 2010 [46]	South Africa	Role of Research in Occupational Therapy Clinical Practice: Applying Action Learning and Action Research in Pursuit of Evidence-Based Practice	Qualitative	Occupational therapy students	Clinical placements	Engagement in small-scale research projects during clinical placements encouraged students’ engagement in research.
Emery 1999 [47]	United States	Use of the Student-Oriented Learning Outline in Research Education Brief report	Mixed-methods investigation of how the Student-Oriented Learning Outline (SOLO) can be used to facilitate student learning in a research course	Occupational therapy students (*n* = 88)	Classroom	Although this use of the SOLO was limited to one institution, experience suggests that over four semesters, the SOLO was effective in helping to structure the research course to facilitate student learning.
Frantz 2009 [48]	South Africa	Perceived Attitudes and Benefits Towards Teaching Evidence Based Practice among Physiotherapy Lecturers at Tertiary Institutions in South Africa	Quantitative within-stage mixed-model approach	Physiotherapy faculty members (*n* = 76) among 8 institutions	Classroom	Although the majority of respondents demonstrated a positive attitude towards teaching EBP, they reported finding it difficult to implement in practice. Despite barriers for teaching EBP, physiotherapy educators are encouraged to be proactive by improving teaching and encouraging teaching based on current, relevant research.
Govender 2019 [49]	South Africa	Making Sense of Knowing: Knowledge Creation and Translation in Student Occupational Therapy Practitioners. Short research report	Pilot study (an exploration of occupational therapy students’ experiences in accessing, implementing and applying current knowledge in their clinical practice as students)	Final-year occupational therapy students (*n* = 24)	Clinical placements	Students seemed to have a general understanding of KT and initiated strategies that are simple and accessible, most of which related to gaining insight into specific intervention in occupational therapy practice.
Halle 2021 [50]	Canada	Building Evidence-Based Practice Competencies among Rehabilitation Students: A Qualitative Exploration of Faculty and Preceptors’ Perspectives	Qualitative 7 focus groups and 1 individual interview with a sample of 24 faculty and 15 preceptors (i.e., clinical instructors)	Faculty and preceptors in occupational therapy, physiotherapy and speech-language	Classroom and clinical placements	Improving collaboration between faculty and preceptors constitutes an essential first step towards more effective EBP training programmes that could be facilitated through online communities of practice or integrated knowledge translation research projects.
Helgøy 2020 [51]	Norway	Research-Based Occupational Therapy Education: An Exploration of Students’ and Faculty Members’ Experiences and Perceptions	Qualitative focus groups	Occupational therapy final year students (*n* = 18) and faculty members (*n* = 12)	Classroom and clinical placements	Research was described as an important aspect of the occupational therapy bachelor programme to ensure necessary competence for offering future best practice. Students expressed a need to be introduced to research early and for higher expectations to be set regarding research. Competence in research methods and the importance of role models were also highlighted.
Hjelle 2008 [52]	Norway	Integrating Evidence-Based Practice in an Occupational Therapy Programme in Bergen	To describe how EBP has been implemented in teaching and to describe research-based knowledge in relation to learning goals	Occupational therapy education	Occupational therapy education	There should be clear EBP competence goals and a progression, and research-based knowledge needs to be highlighted to a larger degree in the curriculum. The occupational therapy programme has a responsibility to ensure students’ learning of EBP skills.
Johnson 2020 [53]	Norway	An Exploration of Evidence-Based Practice Work Files for Occupational Therapy Students during Clinical Placements: A Descriptive Cross-Sectional Study	Quantitative cross-sectional study	Second-year occupational therapy students (*n* = 126)	Clinical placements	Although few students were engaged in EBP, they all reacted positively to it. The students believed that EBP resulted in best clinical care for patients, but they lacked confidence in their own ability to apply EBP.
Lennon 2019 [54]	Ireland	‘The More You Did, the More it Made Sense’: Problem-Based Learning to Improve Early Evidence-Based Practice in an Undergraduate Physiotherapy Professional Programme	Mixed methods	Physiotherapy undergraduate students completing stage 2 of a four-stage, entry-level professional programme	Classroom	Problem-based learning (PBL) is effective at promoting early EBP. Students identified with the interactive, collaborative, and experiential nature of PBL for EBP instruction.
Marais 2019 [55]	Australia	Perceived Enablers and Constraints of Motivation to Conduct Undergraduate Research in a Faculty of Medicine and Health Sciences: What Role Does Choice Play?	Qualitative exploratory descriptive study; semi-structured interviews	Five undergraduate programmes (human nutrition, occupational therapy, physical therapy and speech-language and hearing therapy (the allied health programmes). Students (*n* = 10) and staff members (*n* = 11)	Classroom	To optimize student engagement in research, careful scaffolding of research activities need to be provided, allowing for sufficient choice to enable students to meet their needs for autonomy, competence, and relatedness.
McEvoy 2011 [56]	Australia	Entry-Level Evidenced-Based Practice Training in Physiotherapy Students: Does it Change Knowledge, Attitudes, and Behaviours? A Longitudinal Study	Quantitative cross-sectional study	Entry-level bachelor’s (*n* = 72) and master’s physiotherapy students (*n* = 15)	Classroom	Completion of EBP courses resulted in significant change in all self-reported domains: relevance, sympathy, terminology, practice, confidence and actual knowledge.
Mercer 2002 [57]	New Zealand	Academic Freedom in Physiotherapy Teaching	Opinion piece Grey literature	Physiotherapy education	Classroom	Academic freedom means the teaching staff’s right to be active in research and to have this research inform their teaching. All facets of physiotherapy education should be driven by cutting-edge research and reflect EBP.
Murphy 2019 [58]	Australia	Improving Evidence-Based Practice in Healthcare Courses: A Participatory Action Research Multiple-Case Study	Qualitative grey literature Participatory action research methodology	Faculty members in a bachelor programme for nursing, occupational therapy and physiotherapy	Curriculum for a bachelor programme in nursing, occupational therapy and physiotherapy	All three courses already had strong references to EBP. Identified weakness included a lack of explicit teaching about the meaning, principles, steps and importance of EBP. A relative lack of emphasis on certain EBP steps was also noted, raising concerns about variable EBP education quality across clinical placements. Participatory Action Research seemed to be a highly constructive approach to EBP curriculum improvement.
Olsen 2013 [59]	Norway	Evidence based practice in clinical physiotherapy education: a qualitative interpretive description	Qualitative focus groups: students’ use of EBP in clinical placements	Physiotherapy students (*n* = 16), clinical instructors (*n* = 9) and visiting teachers (*n* = 4)	Clinical placements	Students made an effort to search for research evidence and to apply the knowledge during clinical placements. However, they tended to lean on their clinical instructors and were more eager to gain practical experience than research information. Collaboration between academic and clinical practice is needed to improve competence in EBP among clinical instructors.
Olsen 2014 [60]	Norway	Evidence-Based Practice Exposure and Physiotherapy Students’ Behavior During Clinical Placements: A Survey	Quantitative cross-sectional study	Third year physiotherapy students (*N* = 180) who had recently completed a clinical placement	Clinical placements	An association between the level of EBP exposure and physiotherapy students’ behavior was found for elements such as asking and searching, the ability to search for and critically appraise research evidence, and experience of critical appraisal as a barrier.
Snibsøer 2018 [61]	Norway	Evidence-Based Practice Profiles Among Bachelor Students in Four Health Disciplines: A Cross-Sectional Study	Quantitative cross-sectional study using the Evidence-Based Practice Profile—Norwegian version	Final-year bachelor students in health disciplines from four educational institutions: occupational therapy, physiotherapy, nursing and radiography (*n* = 707)	Classroom	Bachelor students in health disciplines found EBP relevant, but revealed low understanding of EBP terminology, low confidence with EBP skills and low use of EBP in clinical situations. Educational institutions should develop comprehensive curricula where EBP teaching and learning is integrated throughout the entire study programme.
Thomas 2011 [62]	Canada	Evidence-Based Practice: A Review of Theoretical Assumptions and Effectiveness of Teaching and Assessment Interventions in Health Professions	Review	A review of theoretical assumptions and effectiveness of teaching and assessment interventions in health professions		To successfully teach EBP, learning must be embedded within a socially constructed environment using authentic problems. EBP steps included in teaching and case-based methods have great potential for evoking both the knowledge and skills required for evidence-based decision-making.
Thomas 2012 [63]	Canada	Evidence-Based Practice Behaviors: A Comparison Amongst Occupational Therapy Students and Clinicians	Quantitative cross-sectional study	Occupational therapy students (*n* = 51) and occupational therapy clinicians (*n* = 11)	Classroom	The study findings indicate that students showed a greater breadth of knowledge of the process of EBP as taught in the academic programme compared to clinicians.
West 2016 [64]	United Kingdom	Exploring the Barriers and Facilitators to Evidence-Based Practice in Clinical Physiotherapy Education: A Qualitative Study	Qualitative semi-structured interview Grey literature (conference paper)	Final-year physiotherapy students	Clinical placements	The findings from this project support the need for å shift in research culture and the way it is demonstrated to students. Further efforts need to be made to include EBP in the practical curriculum whilst students are on placements.
Wu 2007 [65]	Taiwan	Teaching Evidence-Based Practice to Occupational Therapy Students During Psychiatry Fieldwork: A Curriculum at a University Hospital in Taiwan	Paper that describes the curriculum design of a university hospital in Taiwan employing EBP to future therapists	Occupational therapy students, faculty members and clinical instructors are in focus	Occupational therapy students, faculty members and clinical instructors are in focus	Based on the preliminary results of this curriculum, fieldwork education seems an ideal platform to train occupational therapy students in developing the habit of EBP.
Yousefi-Nooraie 2007 [66]	Iran	Teaching Evidence-Based Practice: The Teachers Consider the Content	Delphi study	EBP teachers (*n* = 51)	Classroom	EBP courses should be simplified and critical appraisal should be limited to studies about therapy, and statistical measures should be reserved for advanced levels. Introduction to systematic reviews and searching for appraised evidence resources should be covered earlier in EBP courses.

### Step 5: summarizing and reporting the results (synthesis of results)

Based on a thematic analysis of the included studies (*n* = 27), we identified strategies to implement research-based education in occupational therapy and physiotherapy bachelor programmes. We also tried to identify where the strategies could be included in the curriculum and when they could be placed in the curriculum. The included studies were classified into four major themes: ‘student engagement in research’ (*n* = 6), ‘curriculum improvement regarding EBP’ (*n* = 2), ‘EBP teaching’ (*n* = 18) and ‘journal club’ (*n* = 1). These strategies are presented in Table 3.

**Table 3:** Strategies used to implement and promote research-based education in occupational therapy and physiotherapy bachelor programmes

First author and year	Study participants/context	Key teaching strategies to implement/promote research-based education	Where to include in curriculum	When placed in curriculum
Bannigang 2009 [41]	Occupational therapy education	EBP teaching	A combination between campus and clinical placement	Throughout the curriculum
Boruff 2011 [42]	Occupational therapy and physiotherapy students	EBP teaching	At campus	Early in the curriculum/first year
Burger 2014 [43]	EBP teachers	Student engagement in research	At campus	Late/final year
Charumbira 2021 [34]	Allied health students (including occupational therapy and physiotherapy students)	Student engagement in research	A combination between campus and clinical placement	Not specified
Chia 2012 [44]	Occupational therapy students	Journal club	A combination between campus and clinical placement	Early in the curriculum/first year
Da Roit 2016 [45]	Physiotherapy students	EBP teaching	A combination between campus and clinical placement	Throughout the curriculum
Du Toit 2010 [46]	Occupational therapy students	Student engagement in research	In clinical placement	Not specified
Emery 1999 [47]	Faculty members (physiotherapy)	Student engagement in research	At campus	Not specified
Frantz 2009 [48]	Occupational therapy students	EBP teaching	Not specified	Not specified
Govender 2019 [49]	Faculty members in occupational therapy, physiotherapy and speech and language therapy	EBP teaching	In clinical placement	Not specified
Halle 2021 [50]	Students and faculty members in occupational therapy	EBP teaching	A combination between campus and clinical placement	Not specified
Helgøy 2020 [51]	Occupational therapy education	Student engagement in research	A combination between campus and clinical placement	Early and throughout the curriculum
Hjelle 2008 [52]	Occupational therapy students	Curriculum strategies for incorporating EBP	A combination between campus and clinical placement	Throughout the curriculum
Johnson 2020 [53]	Physiotherapy students	EBP teaching	A combination between campus and clinical placement	Not specified
Lennon 2019 [54]	Allied health students (including occupational therapy and physiotherapy students) and faculty members	EBP teaching	At campus	Early in the curriculum/first year
Marais 2019 [55]	Physiotherapy students	Student engagement in research	At campus	Not specified
McEvoy 2011 [56]	Physiotherapy education	EBP teaching	A combination between campus and clinical placement	Not specified
Mercer 2002 [57]	Faculty members (nursing, occupational therapy and physiotherapy)	EBP teaching	A combination between campus and clinical placement	Throughout the curriculum
Murphy 2019 [58]	Students, faculty members and clinical instructors in physiotherapy education	Curriculum strategies for incorporating EBP	A combination between campus and clinical placement	Throughout the curriculum
Olsen 2013 [59]	Physiotherapy students	EBP teaching	In clinical placement	Not specified
Olsen 2014 [60]	Occupational therapy students	EBP teaching	In clinical placement	Throughout the curriculum
Snibsøer 2018 [61]	Allied health students (including occupational therapy and physiotherapy students)	EBP teaching	A combination between campus and clinical placement	Throughout the curriculum
Thomas 2011 [62]	EBP teaching and assessment interventions in health professions (a review)	EBP teaching	A combination between campus and clinical placement	Throughout the curriculum
Thomas 2012 [63]	Occupational therapy students and clinicians	EBP teaching	A combination between campus and clinical placement	Not specified
West 2016 [64]	Physiotherapy students	EBP teaching	In clinical placement	Not specified
Wu 2007 [65]	Students, faculty members and clinical instructors in occupational therapy education	EBP teaching	In clinical placement	Late/final year
Yousefi-Nooraie 2007 [66]	EBP teachers	EBP teaching	A combination between campus and clinical placement	Not specified

### Strategies for implementing research-based education across physiotherapy and occupational therapy bachelor programmes

Only one of the included studies focused on research-based education. Two of the included studies investigated curriculum improvement regarding EBP. The majority of the included studies focused on EBP teaching (*n* = 18). Although only one of the studies recommended journal clubs as the main strategy, several of the other studies also recommended journal clubs as a strategy. Findings from the four identified themes are described below.

### Student engagement in research

Six of the included studies focused on student engagement in research [34, 43, 46, 47, 51, 55], whereas only one study had a specific focus on research-based education [51]. In this study [51], emphasizing research in occupational therapy education was perceived as important to ensure students’ future best practice. Moreover, introducing research early in the programme, setting high expectations regarding students’ use of research and achieving skills in research methods were highlighted as necessary. The significance of role models both in clinical placements and among faculty members was described [51]. With regards to the other included studies focusing on research, two had a specific focus on student participation in research projects [46, 55]. In one of these studies [46], occupational therapy students participated in small-scale research projects during their clinical placements and the action learning and action research (ALAR) model contributed to a scholarship of practice where the students, clinical educators and residents of a dementia unit all experienced the value of research. This study [46] indicated that the positive outcomes for students in clinical placements may be complemented by the positive outcomes for practitioners, as they, too, are able to participate in research towards EBP: the authors concluded that a positive approach to research as part of undergraduate training should therefore be emphasized. Further, among students in occupational therapy and physiotherapy programmes, engagement in research might be enhanced if students’ autonomy and influence on factors such as research participation, research topics and groups are structured in the curriculum [55].

Two of the other included studies recommended that student involvement in research could be facilitated by introducing students to systematic reviews [66] and by students themselves to conduct systematic reviews [43]. The authors argued that such experience will give students the opportunity to acquire basic research skills and become clinicians who provide EBP services in clinical practice [43]. Moreover, they recommended that introduction to systematic reviews and searching for appraised evidence resources should be covered earlier in EBP courses [66]. In one study, the use of a Students-Oriented Learning Outline in Research Education (SOLO) was effective in helping to structure the research course to facilitate student learning. A SOLO provides a structure for learning objectives, special instructions and feedback on student progress, as well as flexibility for faculty members to include a variety of instructional methods [47]. Further, 45 research competencies for undergraduate rehabilitation students were identified, in which research methodology and research inquiry/literature reviews are best represented [34]; in this study, five recommendations regarding strategies to develop research and EBP competencies among undergraduates were suggested: collaboration, teaching methods/curriculum design, supervision, assessment and translation of research evidence.

### Curriculum improvement regarding EBP

Two of the included studies, conducted in Norway and Australia, focused on improving the curriculum regarding EBP [52, 58]. Here, based on evaluation of EBP in healthcare courses, participatory action research methodology was a recommended approach for curriculum improvement [58]. Moreover, authors argued that there should be clear EBP competence goals and a progression, and research-based knowledge needs to be highlighted to a larger degree in the curriculum [52].

### EBP teaching

The majority of the included studies focused on EBP teaching [41, 42, 45, 48–50, 53, 54, 56, 57, 59–66]. Several of these studies recommended teaching and learning EBP in clinical placements in addition to the classroom [49, 53, 56, 57, 59, 64–66]. Findings indicate that EBP in clinical placements can serve as an ideal platform for collaboration between students, occupational therapy faculty members and clinical instructors. Further, EBP teaching and workshops during clinical placements is necessary and journal clubs have been suggested as a way to foster collaboration [65]. EBP teaching should be integrated in routine clinical practice and be considered a real-time continuous and flexible process [66].

#### Collaboration with clinical practice regarding EBP

Four of the included studies focused on collaboration with clinical practice regarding EBP [50, 53, 59, 64]. Further efforts to include EBP in clinical curricula was emphasized by West [64], who highlighted the importance of collaboration between students and clinical instructors and the impact of attitudes held by clinicians. The use of discussion groups in clinical placements between students and supervisors and the use of research in treatment plans have been recommended in physiotherapy education [59]. In occupational therapy education, Johnson [53] highlighted that EBP teaching and its associated assignments should include the active involvement of both students and clinical instructors. Moreover, faculty members should involve and collaborate with clinical instructors, so that students can present and discuss the results of research articles and implement EBP in clinical placements: journal clubs might represent one way to foster this kind of collaboration between students and clinical instructors [53]. Collaboration with practice to achieve more effective EBP training—such as through online communities of practice or integrated knowledge translation research projects— has also been suggested [50].

#### EBP teaching strategies

Regarding EBP teaching in classroom, one study recommended more time for EBP, journal club activities and a formal assessment in the first year [45]. McEvoy [56] argued that an EBP course in combination with clinical placements resulted in significant changes in students’ self-reported domains regarding EBP. There should be a link between academic subjects and practice: two of the included studies had a focus on physiotherapy faculties teaching [48, 57], where Frantz [48] argued that physiotherapy educators should be proactive by improving teaching and encouraging teaching based on current, relevant research. The authors of [61] argued that physiotherapy teaching staff have the right to be active in research and to have this research inform teaching, and that all facets of physiotherapy education should be driven by cutting-edge research and reflect EBP. One of the studies [65] focused on problem-based learning (PBL) as an approach to EBP instruction, concluding that PBL promotes improvement in EBP skills, communication, teamwork and EBP team self-efficacy. Further, scaffolded learning for early stage EBP competence is required and a PBL approach allowed greater emphasis on research processes. EBP training should be emphasized throughout the curriculum [60, 61], allowing repetition, consolidation and application of EBP knowledge and skills [61]. The importance of teaching all EBP steps has been highlighted in two of the studies [62, 63] and case-based methods were suitable for students EBP learning [62]. One of the studies [56] highlighted that professional thinking should be taught as an overarching topic within curricula where EBP is included. Collaboration between the faculty member and the librarian was a success in teaching students EBP skills [42]. Strategies for students to be able to translate knowledge to practice have been suggested, including discussions with peers and clinicians, hands-on practice in the application of knowledge in research projects and the use of knowledge tools and social media [49].

### Journal club

One of the included studies had a specific focus on the use of journal clubs in occupational therapy education [44]. In this study, the journal club was included in the first year of the programme and the authors recommended the use of journal club in small groups of approximately 12 students with a subgroup of 3 students [44]. The authors highlighted participation in journal clubs at an early stage as a foundation for engagement with professional literature and the recognition of the importance of EBP. Five of the other included studies also recommended use of journal clubs in addition to other teaching strategies [45, 49, 52, 53, 65]. Journal club was not a part of their study design, however. One of these studies highlighted that student- and clinician-driven journal clubs are recommended among occupational therapy students related to knowledge translation [49]. The use of journal clubs has also been suggested to strengthen the cooperation between occupational therapy students, scientific staff and clinical instructors [52, 53, 65]. A recommendation from one of the studies is that, among physiotherapy undergraduates, the use of journal clubs should be increased in the curriculum [45].

### Where and when to include the strategy in the curriculum

Five of the included studies [42, 43, 47, 54, 55] recommended that the teaching strategy be included on campus, 6 [46, 49, 59, 60, 64, 65] recommended that it be included in clinical placements and 15 [34, 41, 44, 45, 50–53, 57, 58, 61–63, 66, 67] recommended a combination between campus and clinical placements. One of the studies [48] did not specify this aspect. Four of the included studies [42, 44, 51, 54] recommended including the teaching strategy early in the curriculum, two [43, 65] recommended its inclusion in the final year/late stages of the curriculum and eight [41, 45, 52, 57, 58, 60–62] proposed that it be included throughout the curriculum. Thirteen of the studies did not specify when the teaching strategy should be included in the curriculum.

## Discussion

In this scoping review, the purpose was to identify studies reporting on research-based education in undergraduate occupational therapy and physiotherapy curricula to document the current state of knowledge. Further, an objective was to map factors that reflect and support the implementation of research-based education in undergraduate occupational therapy and physiotherapy programmes. Based on a systematic and thorough process, 27 studies were included. Of these studies, 16 were published within the last 10 years, indicating an increased perceived importance of training students to be critical users of research. The current state of knowledge indicates that studies investigating research-based education are scarce. The majority of the studies focused on aspects of EBP; however, EBP and research-based education are different concepts. The goal of EBP is to use the highest possible quality of knowledge in providing care to ensure the greatest impact on patients’ health status and healthcare outcomes [26]. EBP entails making professional decisions based on systematically retrieved research evidence, experiential knowledge and patient preferences in a given situation [4, 68]. By contrast, research-based education has a wider focus and is more concerned with creating a learning environment where academics and students teach and learn through inquiry and research [1]. Further, research-based education consists of both research-led and research-based teaching [1]. Nevertheless, EBP has relevance for discussions regarding research-based education when it comes to using research evidence in professional practice. With regards to implementing and supporting research-based education in occupational therapy and physiotherapy programmes, we identified the following four strategies: ‘student engagement in research’, ‘curriculum improvement regarding EBP’, ‘EBP teaching’ and ‘journal club’. The majority of the included studies recommended implementing the suggested strategy in a combination of classroom and clinical placements and throughout the curriculum. Since 13 of the included studies did not specify where to include the strategy in the curriculum, it is difficult to make conclusions regarding the timing of each of the suggested strategies. The findings discussed below are related to the strategies most frequently identified in our thematic analysis and discussed in light of previous research, conceptual frameworks and curriculum strategies regarding research-based education.

### Student engagement in research

Studies included in the current review highlight the importance of emphasizing research in the education programme to ensure that students have the skills to provide future best practice [51] and develop students research skills and competence [34, 43, 51, 66]. This may be accomplished by participation in research projects [46, 55]. Previous research among occupational therapy and physiotherapy students has identified positive attitudes towards research, particularly related to reading research literature and applying research findings to improve practice [69]. Learning research skills to improve practice and benefit healthcare outcomes has also been highlighted as important in nursing education, where student–faculty collaborative projects have been recommended [70, 71]. This is in agreement with previous research in medical education, where developing students research skills has been highlighted as important for ensuring that students make informed decisions in their future practice [72]. Moreover, student participation in research has improved their scientific productivity, knowledge, interest and attitudes towards research [73, 74]. Based on the reviewed literature relating to occupational therapy and physiotherapy education programmes, it appears important that students’ engagement in research clearly links with professional practice. Moreover, students need to develop research skills suitable to provide future best practice; one approach is by introducing research methods to students early and facilitating participation in inquiry-based activities throughout the education programme. Studies investigating inquiry-based activities and students’ engagement with research appear to be useful: these will hopefully expand in number and scope, as more knowledge regarding students’ experiences and learning outcomes from such activities is needed.

### Curriculum improvement regarding EBP

Regarding curriculum, clear EBP competence goals and a progression has been suggested, and research-based knowledge should be highlighted to a larger degree [52]. This is in line with policy documents, such as those from the World Federation of Occupational Therapists [75] and World Physiotherapy [76]. These documents recommend a curriculum that includes critical thinking, problem-solving, EBP, research and lifelong learning [75], and encourage and support a curriculum which is evidence-based/informed and develops students’ research skills [76]. Moreover, physiotherapists’ education framework emphasizes EBP, literature searches and reviews, research methodologies and research in physiotherapy education [77]. This is in agreement with the AMEE Guide [78], which centres on curriculum design related to developing undergraduates’ research skills in medical education. In this guide, it is highlighted that medical students must understand research methods and the benefits that research brings to their profession. Students’ active participation in research activities is suggested, and adopting a student-centred approach in the curriculum may facilitate developing students research skills [78]. Here, suitable methods may include project-based learning, case-based learning, inquiry-based learning or PBL methodologies within the curriculum design [78]. The role of accreditation will also have an impact on the integration of research in the curriculum, as the expectations of both faculty members’ and students’ research skills may increase [23]. Further, accreditation has the potential to improve health-care outcomes, with its ability to influence and standardize the quality of education programmes [23]. Strengthening the research integration in curricula had a positive effect on research-related student learning outcomes among first-year medical students [79].

Few of the included studies in our review focused on curriculum design or improvement; instead, the focus was mainly on EBP. However, previous studies have focused on strategies to support faculty in teaching students research and inquiry [20, 21]. It has been argued that engaging students in research and inquiry early on and ensuring a progression in research and inquiry in the education programme is important [20]. Further, ensuring systematic thinking and coherence in the curriculum regarding how students can obtain the needed skills to engage with research has been highlighted [21]. Moreover, inquiry-based learning seems to be one of the key aspects of research-based education, and student-active learning can be beneficial for developing students’ analytical and critical thinking skills [21]. Huet [1] focused on implementing research-based education in curricula and recommended four actions to succeed. First, the institution must have clear guidelines for embracing a research-based education model at all levels of the study programme. Second, the institutional policies must take into account the time needed to conceptualize, plan and deliver research-based and research-led teaching approaches. Third, the importance of facilitating a culture of research and teaching must be highlighted as two integrated activities. Fourth, academics must be provided with pedagogic support, preparing them to develop learning and teaching strategies that are sustained in research-led or research-based approaches in early stages in the curriculum. Based on these recommendations, it seems beneficial to embrace a research-based education model in the education programme and to integrate research with teaching in the organization of that programme. Related to occupational therapy and physiotherapy curricula the emphasis on research should be increased to ensure that students have research skills that can benefit their professional practice and healthcare outcomes. Learning and teaching strategies may be designed in agreement with research-based teaching and research-led teaching [1].

### EBP teaching

The most frequently identified strategies in our review were related to EBP teaching. Findings from these studies indicate that the teaching and learning of EBP should be emphasized in clinical placements [49, 53, 56, 57, 59, 64–66] and in collaboration with clinical practice [50, 53, 59, 64]. Further, early EBP education led to adequate knowledge, confidence and positive attitudes [56]. Teaching should be based on cutting-edge research and reflect EBP [57], and educators should be proactive by improving teaching and encouraging teaching based on current research as well as being active in research themselves [48]. Having research inform teaching and educators be active in research projects coincides with research-led teaching, as described by Huet [1]. However, Huet emphasized that educators do not need to be active researchers or perform cutting-edge research, but they *do* need to be engaged in a scholarly manner within their field [1]. Moreover, findings from studies included in our review indicate that all the EBP steps should be included in teaching [62, 63]: this is in agreement with previous research that indicates that most EBP educational interventions focus on teaching only some of the EBP steps [80]. Further, multifaceted approaches including lectures, tutorials, workshops and journal clubs have also been found to be beneficial when teaching EBP to health students from programmes such as medicine, physiotherapy and occupational therapy [32, 33]. Among professional bachelor degree healthcare programmes, research courses, workshops and collaboration with clinical practice are recommended as methods for teaching EBP [28].

Summarizing the above, collaboration with clinical practice and emphasizing teaching and learning of EBP in clinical placements seems to be beneficial in occupational therapy and physiotherapy education programmes; further, multifaceted approaches in teaching EBP should be emphasized, and teaching activities could be based on cutting-edge research and reflect EBP.

### Journal club

Findings from studies included in our review indicate that journal clubs are a well-suited means to increase students’ engagement with professional literature and to recognize the importance of EBP [44]. Journal clubs may be suitable as a method for students to implement research skills in clinical placements and to facilitate collaboration with clinical instructors [52, 53, 65]. Moreover, journal clubs have been proposed to support knowledge translation [49], and more frequent use of journal clubs in undergraduate curricula has been advocated [45]. Similar to the findings in our review, previous studies have argued that journal clubs can assist students’ EBP learning process [81] and add to their EBP skills, particularly regarding students’ ability to search the literature and to comprehend and appraise research articles [82]. However, other studies did not find improvement in critical appraisal skills from students’ participation in journal clubs [83–85]; nevertheless, students have reported an overall positive experience from participating in them, especially with a view towards discussing research utilization [86]. Further, journal clubs can be suitable as a learning and collaboration method between education and practice [87, 88]. Previous reviews among medical students, as referenced by Young et al. [33], have described a positive effect of journal clubs as a method for teaching EBP [83–85, 89].

We did not identify many studies investigating journal clubs among occupational therapy and physiotherapy students, and more studies are needed. In view of the above, it appears that implementing journal clubs in occupational therapy and physiotherapy education can offer advantages, such as improving students’ searching and reading skills, helping them stay up-to-date and facilitating research utilization in clinical placements and future practice. Journal clubs can be introduced in the first year as a part of a research or EBP course, facilitated by academic staff.

### What can be learned from this scoping review?

Based on the findings from our review, knowledge regarding research-based education and strategies to support the implementation of research-based education in occupational therapy and physiotherapy curricula are scarce. EBP has been emphasized to a greater degree in studies in these programmes, and more knowledge is needed to implement research-based education in curricula. Only one of the included studies explored research-based education [51]. However, some elements of both research-led and research-based teaching were described in the included studies. Emphasizing students’ active involvement in research projects, conducting systematic reviews and participation in journal clubs may be perceived as a practical means of implementing research-based teaching. Similarly, research-led teaching is exemplified by educators who utilize cutting-edge research findings and use their own research to support their teaching.

Research training is important for all health science professions [34], and in professional programmes such as occupational therapy and physiotherapy it is important that the emphasis on research is linked to professional practice and implementing research as a part of EBP. As described earlier, EBP and research-based education are different concepts. However, EBP has relevance for discussions regarding research-based education when it comes to using research evidence in professional practice. As highlighted by Brew, ‘some of the best examples of undergraduate students engaging in inquiry processes in order to learn come from professional areas. As professions become increasingly evidence based, valuing the use of research as an integral part of professional practice, students are also increasingly being required to engage in inquiring into aspects of practice in order to learn’ ([90] , p. 19). Creating a research-based learning environment thus has benefits for occupational therapy and physiotherapy students, as they can learn how to become critical thinkers and lifelong learners and to generate discipline-enriching knowledge [1]. These skills are important for their future professional practice.

### Implications for educational practice

Based on the included studies in our review, we recommend that students’ learning of research methods should be prioritized. More methodological competence seems to be needed for students to be able to read and understand research articles—it is therefore foundational for undergraduate research training [34, 51]. Research should be introduced early in the education programme, including introducing students to systematic reviews and searching for appraised evidence [66]. Involving students in research projects might be a good learning arena, as it may encourage students’ engagement in research and promote collaboration with clinical practice [46, 55]. Students conduct systematic reviews could be emphasized, as this might provide basic research skills and encourage the use of EBP in future clinical practice [43, 66].

The majority of the strategies identified in this review were related to ‘EBP teaching’. Based on the included studies, we recommend that the teaching and learning of EBP should take place in clinical placements in addition to classroom teaching [49, 53, 56, 57, 59, 64–66]. Further, all EBP steps should be included in teaching where case-based methods may be a suitable approach [62, 63]. It is recommended that educators should base their teaching on current relevant research and be active researchers themselves [57]. Journal clubs should be emphasized in the curriculum as it can be a foundation for engagement with professional literature and the recognition of the importance of EBP [44]. Journal clubs have also been recommended as a means for cooperation between occupational therapy students, scientific staff and clinical instructors [52, 53, 65]. Moreover, there should be increased focus on research-based knowledge in the curriculum, and progression needs to be ensured [52]. To succeed with implementing research-based education, awareness of the research-based education model should be increased in education programmes, and different teaching and learning strategies should be explored [1]. Elements of research-based education should be reinforced at all level of the study programme, and introduced early [1]. Relevant organizational units, such as faculties and departments, must take responsibility for raising students’ and educators’ awareness of the importance of linking research and education, and how the implementation and use of research in education can be promoted in different ways [1]. In professional programmes such as occupational therapy and physiotherapy, research-based education will preferably be designed such that learning and teaching activities are relevant to the specific fields of professional practice [22, 51].

### Strengths and limitations

A strength with this scoping review is that it complies with the key steps outlined in the Arksey and O’Malley 2005 [35] framework, and further developed by Levac et al. 2010 [36] and Khalil et al. 2016 [37], to systematically select and summarize existing literature focusing on research-based education. We also used the JBI manual for conducting scoping reviews to ensure a systematic methodology [38]. Our scoping review presents an overview of existing literature describing research-based education in occupational therapy and physiotherapy bachelor programmes. However, some limitations need to be addressed. There is a possibility that relevant studies were included in this review, although several databases and grey literature were searched. The quality of the included studies was not assessed as a part of this scoping review, as according to Arksey and O’Malley [35] there is no attempt made to present a view regarding the ‘weight’ of evidence in relation to particular interventions or policies. This is because the scoping study does not seek to assess quality of evidence and consequently cannot determine whether particular studies provide robust or generalizable findings.

## Conclusion

This scoping review has provided an extensive overview of literature describing research-based education in occupational therapy and physiotherapy programmes. However, only a few of the included studies in this review had specifically investigated aspects of research-based education. The most frequently identified strategies in our review were related to EBP teaching. Overall, our findings show that ensuring students’ competence in research methods is necessary for students to be able to read and understand research articles, which are important foundational skills in undergraduate research training. Journal clubs may give students a foundation for engaging with research literature. Moreover, students’ basic research skills may be facilitated through their involvement in research projects and by conducting systematic reviews. Cooperation with clinical practice is important in occupational therapy and physiotherapy education. Faculty members should be proactive by improving teaching based on current, relevant research, and the awareness of research-based education should be increased among both faculty members and students.

Based on our findings, research-based education appears to be a new and relatively unexplored research field in the context of occupational therapy and physiotherapy education programmes, and we argue that more studies investigating research-based education in these programmes are needed. As so few of the included studies focused on faculty members, more studies among faculty members—concerning their views, experiences and practices related to research-based education—are needed. Moreover, future research could investigate viable and effective strategies for teaching research methods to undergraduate healthcare students, students’ learning outcomes regarding research methods and students’ research skills and involvement in research projects. Further, cooperation between education programmes and clinical placements regarding research implementation should be explored. Scant studies investigating curriculum design for integrating research seem to exist, and more such studies are needed in this area. Future studies may benefit from investigating curriculum design in accordance with research-based education curriculum and based on the policy documents concerned with implementing research and developing students’ research skills.

## Supplementary Information

**Additional file 1.**

## Data Availability

Data sharing is not applicable to this article as no datasets were generated or analyzed during the current study.
