# Supplementary material for: Research-based education in undergraduate occupational therapy and physiotherapy education programmes: a scoping review
Source: BMC Med Educ. 2022 May 11;22:358. doi: 10.1186/s12909-022-03354-2 (PMC9097417; doi:10.1186/s12909-022-03354-2)
Supplement: Supplementary file 1 — Additional file 1. [file 12909_2022_3354_MOESM1_ESM.docx]

**Appendices**

**Appendix 1: Template study details and characteristics and results extraction instrument**

**Review title:** Research-based education in undergraduate occupational therapy and physiotherapy education programmes: a scoping review

**Research question:** The purpose was to identify studies reporting on research-based education in undergraduate occupational therapy and physiotherapy curricula to document the current state of knowledge. Further, an objective was to map factors that reflect and support the implementation of research-based education in undergraduate occupational therapy and physiotherapy educational practice.

**Inclusion criteria (PCC)**

**Population:** Studies reporting on students and/or academic staff in occupational therapy and physiotherapy bachelor programmes.

**Concept:** Studies with a primary focus on research-based education.

**Context:** A typical educational setting, e.g., classroom teaching, clinical placement or simulation training.

**Exclusion criteria:** Literature including bachelor students and/or academic staff in health disciplines other than occupational therapy and physiotherapy were excluded. Studies including students from master’s degree programmes were also excluded. Moreover, studies including occupational therapy and physiotherapy clinicians and studies with other languages than English, Danish, Norwegian or Swedish were excluded.

**Study details and characteristics extraction:**

**First author**

**Year of publication**

**Title**

**Country of origin**

**Study design**

**Study participants**

**Context**

**Main study findings**

**Results extraction (Level 2)**

**Strategies used to implement and promote research-based education**

**Recommendations regarding when** (early, late or throughout the curriculum) **and where to include in the curriculum** (clinical placements, classroom or a combination).
